# NBD delivery improves the disease phenotype of the golden retriever model of Duchenne muscular dystrophy

**DOI:** 10.1186/2044-5040-4-18

**Published:** 2014-10-23

**Authors:** Joe N Kornegay, Jennifer M Peterson, Daniel J Bogan, William Kline, Janet R Bogan, Jennifer L Dow, Zheng Fan, Jiahui Wang, Mihye Ahn, Hongtu Zhu, Martin Styner, Denis C Guttridge

**Affiliations:** 1 Department of Pathology and Laboratory Medicine, School of Medicine, University of North Carolina, Chapel Hill, NC 27599, USA; 2 Department of Neurology, School of Medicine, University of North Carolina, Chapel Hill, NC 27599, USA; 3 Department of Psychiatry, School of Medicine, University of North Carolina, Chapel Hill, NC 27599, USA; 4 Department of Biostatistics, Gillings School of Global Public Health, University of North Carolina, Chapel Hill, NC 27599, USA; 5 Department of Computer Science, University of North Carolina, Chapel Hill, NC 27599, USA; 6 The Gene Therapy Center, School of Medicine, University of North Carolina, Chapel Hill, NC 27599, USA; 7 Department of Molecular Virology, Immunology, and Medical Genetics, The Ohio State University, Columbus, OH 43210, USA; 8 Department of Veterinary Integrative Biosciences, College of Veterinary Medicine, Texas A&M University, Mail Stop 4458, College Station, TX, USA; 9 The Ohio State University College of Medicine, 460W. 12th Avenue, Columbus, OH 43210, USA

## Abstract

**Background:**

Duchenne muscular dystrophy (DMD) is caused by mutations in the dystrophin gene and afflicts skeletal and cardiac muscles. Previous studies showed that DMD is associated with constitutive activation of NF-κB, and in dystrophin-deficient *mdx* and utrophin/dystrophin (*utrn*^*-/-*^*;mdx*) double knock out (dko) mouse models, inhibition of NF-κB with the Nemo Binding Domain (NBD) peptide led to significant improvements in both diaphragm and cardiac muscle function.

**Methods:**

A trial in golden retriever muscular dystrophy (GRMD) canine model of DMD was initiated with four primary outcomes: skeletal muscle function, MRI of pelvic limb muscles, histopathologic features of skeletal muscles, and safety. GRMD and wild type dogs at 2 months of age were treated for 4 months with NBD by intravenous infusions. Results were compared with those collected from untreated GRMD and wild type dogs through a separate, natural history study.

**Results:**

Results showed that intravenous delivery of NBD in GRMD dogs led to a recovery of pelvic limb muscle force and improvement of histopathologic lesions. In addition, NBD-treated GRMD dogs had normalized postural changes and a trend towards lower tissue injury on magnetic resonance imaging. Despite this phenotypic improvement, NBD administration over time led to infusion reactions and an immune response in both treated GRMD and wild type dogs.

**Conclusions:**

This GRMD trial was beneficial both in providing evidence that NBD is efficacious in a large animal DMD model and in identifying potential safety concerns that will be informative moving forward with human trials.

## Background

Duchenne muscular dystrophy (DMD) is an X-linked recessive disease, in which mutations in the gene coding for the protein dystrophin lead to progressive degeneration of skeletal and cardiac muscles [1-3]. Glucocorticoids, such as prednisone, are the current standard of care for DMD [4,5], but in spite of clinical benefits, treatment must often be discontinued due to side effects [6]. This has prompted use of many different glucocorticoid protocols and development of alternative pharmacologic approaches directed at specific pathogenetic mechanisms with fewer complications.

Treatments targeting NF-κB signaling are of particular interest because glucocorticoids exert their effects, in part, by blocking this pathway [7]. Studies have also shown that NF-κB signaling is activated in DMD patients and exacerbates muscle lesions and dysfunction in DMD mouse models [8,9]. NF-κB signaling occurs in response to factors such as inflammatory cytokines [10]. These stimuli activate the inhibitor of kappa B kinase (IKK) complex, which consists of two catalytic subunits (IKKα and IKKβ) and a regulatory subunit (IKKγ/NEMO) [11]. In resting cells, the inhibitor protein, IκB, binds and maintains NF-κB in an inactive complex in the cytoplasm. Upon activation, IKK phosphorylates IκB, leading to its ubiquitination and subsequent degradation by the 26S proteasome. This in turn allows NF-κB to translocate to the nucleus and cooperate with basal transcription factors to enhance transcription of its target genes.

The Nemo Binding Domain (NBD) peptide is a specific inhibitor of NF-κB that functions by binding to sequences within IKKα and IKKβ that permit interaction with NEMO [12]. By effectively inhibiting assembly of the IKK complex, NBD prevents activation of NF-κB. Inhibiting NF-κB signaling with NBD reproducibly alleviates dystrophic histopathologic lesions and improves muscle function in DMD mouse models. Specifically, NBD-treated dystrophin-deficient *mdx* mice have reduced inflammation and injury, as well as enhanced regeneration and function in skeletal muscles [9,13]. In addition, NBD has been shown to prevent cardiac dysfunction in utrophin/dystrophin (*utrn*^*-/-*^*;mdx*) double knock out (dko) mice [14]. Besides NBD, other strategies to reduce NF-κB signaling in dystrophic or injured mice, including the use of muscle derived stem cells deficient in one copy of the p65/RelA NF-κB subunit [15], or with gene therapy using viral interference of IKK activation [16], have provided additional evidence that NF-κB inhibition is advantageous for treating/repairing injured muscles. Altogether, these studies have been encouraging, indicating that NF-κB inhibition may be a viable avenue for treating DMD.

Golden retrievers with muscular dystrophy (GRMD) have a spontaneous mutation in the dystrophin gene and develop phenotypic features typical of DMD [17-19]. Unlike the *mdx* mouse, which exhibits a mild and stable phenotype when compared to the progressive disease in DMD boys, affected GRMD dogs undergo progressive fatal disease. This phenotypic similarity suggests that studies in dystrophic dogs may effectively predict relevant disease mechanisms and therapeutic efficacy. Indeed, the GRMD model has been used increasingly in preclinical trials of various therapeutic modalities, including genetic, cellular, and pharmacologic approaches [20].

In the current study, we administered NBD intravenously to GRMD dogs, employing a treatment protocol and biomarkers used previously to establish both benefits and potential deleterious effects of prednisone [21]. Consistent with observations in mice, we found that NBD treatment improved function and ameliorated muscle histopathologic lesions in GRMD dogs, supporting the use of NBD as a therapeutic for DMD.

## Methods

### Intravenous dosing in mice

*Mdx* mice (C57BL/10ScSn-*DMD*^*mdx*^/J) were purchased from The Jackson Laboratory and housed in the animal facility (University Laboratory Animal Resources, ULAR) at The Ohio State University under conventional conditions with constant temperature and humidity, and fed with standard diet. Treatment of mice was performed between 5 and 7 weeks of age as earlier described [13], and in accordance with the guidelines of the Institutional Animal Care and Use Committee at The Ohio State University. Efficacy of NBD was assessed comparing four groups (n = 5 each) of *mdx* mice treated either with vehicle or 3, 2, or 1 × per week with NBD by intraperitoneal (IP) delivery. Two separate groups (n = 10) were dosed subcutaneously (SQ) with vehicle or NBD, and finally two groups (n = 5) were treated with vehicle or NBD by intravenous (IV) delivery. Vascular access ports (VAP) were placed subcutaneously over the dorsal torso and a catheter was surgically inserted into the jugular vein. The catheter was kept clear by a pre- and post-wash with heparin.

### Canine experimental design

All dogs were produced in a colony at the University of North Carolina at Chapel Hill (UNC-CH) and were used and cared for according to principles outlined in the National Research Council Guide for the Care and Use of Laboratory Animals. The UNC-CH Institutional Animal Care and Use Committee approved procedures.

The GRMD disease phenotype was initially determined based on elevation of serum creatine kinase and confirmed by PCR. Two cohorts of GRMD dogs were treated with a 4-month course of NBD (10 mg/kg, IV) (American Peptides; Sunnyvale, CA, USA) [13], beginning at approximately 2 months of age. The first cohort included four GRMD (Wasabi, Pepper, Hiver, and Automne) and two wild type (Cumin and Fennel) dogs, while the second cohort included two GRMD (Peach and Kiwi) and one wild type (Mango) dog. Results were compared with those collected from 10 untreated GRMD dogs (Cilantro, Lyle, Napoleon, Summer, Jane, Cosmo, Dorothy, Toto, Hickory, and Zeke) and eight age-matched wild type littermates (Oregano, Parsley, Kip, Pedro, Pinkman, Saul, Heisenberg, and Tuco) through a parallel, but separate, natural history study in which functional, magnetic resonance imaging (MRI), and pathologic data were collected.

### NBD preparation and administration

NBD peptide (TALDWSWLQTE) fused to an Antennapedia protein transduction domain [9] was generated using an ABI 430A solid-phase peptide synthesizer (Applied Biosystems, Foster City, CA, USA) as previously described [13]. NBD solutions (10 mg/mL) for the canine studies were prepared weekly. Needed volumes were calculated based on the current dog body weights, plus estimated weekly gain averages. Compound was weighed on a laboratory balance to the nearest 0.1 g and reconstituted in sterile water. Solution was then sterile-filtered through 0.22 μm filters into a sterile fluid administration bag and refrigerated at 4°C until use. Daily administration volumes (total volume for all dogs perfused for the day) were drawn up into a sterile 20 or 60 mL syringe, fitted with an intravenous tubing extension set, and loaded into a syringe pump (Medfusion™ 3500 Syringe Pump; Smiths Medical, St Paul, MN, USA). Prior to perfusion, dogs were premedicated with butorphanol (0.4 mg/kg, IM); once infusion reactions were seen, diphenhydramine (2.0 mg/kg, SQ) was also given. Heart and respiratory rate, mucous membrane color, capillary refill time, and body temperature were monitored throughout the perfusion. Approximately 10 to 20 min after premedication, intravenous catheters (22 to 24 gauge) were placed sterilely into either the cephalic or saphenous vein. The syringe pump was initially programed to administer the calculated volume over 10 min, but this was extended to 30 min when reactions were seen. Blood pressure was recorded prior to start of perfusion, at 5-min intervals throughout perfusion, and post-perfusion (Cardell^®^ 9405 Multiparameter Monitor, Midmark Corporation, Versailles, OH, USA). Dogs were monitored for adverse reactions throughout the perfusion and for up to 30 min after completion.

### Pharmacokinetic (PK) measurements

PK studies were performed at Sinclair Research Center (Auxvasse, MO, USA). Whole blood was collected from normal mice (0.5 to 1.0 mL; n = 3/time point) and beagle dogs (3.0 mL; n = 3/group) at 0, 0.08, 0.25, 0.5, 1, 2, 4, 8, and 24 h following IV dosing with NBD at 2 and 10 mg/Kg and transferred into pre-labeled tubes containing EDTA as an anticoagulant. Plasma was prepared and shipped overnight to Frontage Labs (Malvern, PA, USA) for PK evaluations. PK calculations were performed using WinNonlin Professional software from plasma concentration, and parameters were determined directly from the plasma concentration.

### Tibiotarsal joint (TTJ) force measurements

For all tests, dogs were anesthetized (premeds: acepromazine maleate (0.02 mg/kg), butorphanol (0.4 mg/kg), and atropine sulfate (0.04 mg/kg); masked/intubated; and maintained with sevoflurane). To assess force and eccentric contraction decrement (below), dogs were positioned in dorsal recumbence in a custom-made stereotactic frame that aligns the tibia parallel to the table at a 90° angle to the femur. The angle at which maximal joint torque is generated during isometric contractions has been termed the optimal joint angle [22], which is analogous to the optimal fiber length (Lo) for individual muscle force measurements. Our choice of 90° as the optimal joint angle was based on studies in which torque was measured over a range of angles [23]. The length-tension relationship was not shifted for normal versus GRMD dogs. TTJ flexion and extension torque (N-m) was measured by a rapid-response servomotor/force transducer (model 310B LR, Aurora Scientific, Inc., Aurora, ON, Canada) controlled by a PC using custom LabView software [24,25]. Either the common peroneal (TTJ flexion) or tibial (TTJ extension) nerve was stimulated using paired stimulating and reference 27-gauge monopolar electrodes placed just distal to the fibular head (common peroneal nerve) or within the gastrocnemius muscles (tibial nerve), respectively. As a result, the paw of the distal pelvic limb pulled (flexion) or pushed against (extension) a pedal affixed to a transducer, providing a measure of isometric torque. Supramaximal 150 V, 100 μs pulses were applied (Model S48 Solid State Square Wave Stimulator; Grass Instruments, Quincy, MA, USA) in a tetanic run of 250 pulses (50/s). The site of contact for the paw with the lever (moment arm) was estimated to be 75% of the distance between the point of the hock and the distal digit. Torque (Newton-meters) was divided by the moment arm (meters) to convert to force (Newtons).

### Eccentric contraction decrement (ECD)

Eccentric contractions were induced by stimulating the peroneal nerve using square wave pulses of 100 μs duration in a tetanic run for 1 s at a frequency of 50 Hz while simultaneously extending the TTJ with a servomotor (Aurora Scientific, Aurora, ON, Canada) [24-26]. The contraction was held isometric at the optimal joint angle [22], expressed as Lo here (see discussion above), for the first 900 ms. For the final 100 ms, the muscles of the cranial tibial compartment were stretched by the servomotor at 0.7 Lo/s, such that the muscles were displaced to 107% of Lo. Thus, the muscles of the cranial tibial compartment were repeatedly stretched to induce mechanical damage. Three sets of 10 stretches for a total of 30, each set separated by 4 min, were performed. Contraction-induced injury was quantified by the force (torque) deficit (Fd) using the following equation: Fd = (Maximal isometric tetanic force (Po) before stretch - Po after stretch/Po before stretch) × 100.

### Joint angles

We previously reported that 6-month-old GRMD dogs have abnormally acute (contracted) TTJ angles while positioned in dorsal recumbence for force measurements [27,28]. Other investigators have subsequently described methods to measure joint angles at maximal flexion and extension, with associated range of motion, in wild type dogs [29,30]. The method of Jaegger *et al.*[29] is now utilized to measure pelvic limb tibiotarsal (hock, ankle), stifle (knee), and coxofemoral (hip) joint angles for ongoing natural history and preclinical trials in our laboratory. In each case, dogs were anesthetized (above) and positioned in lateral recumbence. Angles at rest, maximal flexion, and maximal extension were measured. To objectively characterize the cranioventral shift of the pelvis typically seen in GRMD dogs, we also measured the pelvic angle formed by two lines extending cranially from the tuber ischium, one drawn parallel to the lumbar spine and the other extending to the midpoint of the tuber coxae.

### Magnetic resonance imaging

Studies were done on a Siemens 3 T Allegra Head-Only MRI scanner with a circular polarization (CP) head coil or Siemens 3 T Tim Trio Whole-Body MRI scanner with a 32-channel body coil at the UNC-CH Biomedical Research Imaging Center (BRIC) [31,32]. Dogs were anesthetized (above), placed on an MRI gantry in the sternal (prone) position with the pelvic limbs extended, and positioned in the coil centered at the midpoint of the femur. The proximal pelvic limbs from the coxofemoral joint to the stifle were imaged bilaterally. Scans were completed using a published protocol [31,32]. T2-weighted image sequences without (T2w) and with fat saturation (T2fs) were acquired using a variable-flip-angle turbo spin echo (TSE) sequence. The time between the excitation pulse and the center of k-space was 400 ms. Importantly, the contrast was not determined only by the TE (400 ms), but also by the flip angle evolution scheme. Although a traditional TSE sequence would have very little signal at 400 ms, the variable flip angle sequence is similar in principle to hyper-echo. The hyper-echo reduces the specific absorption rate (SAR), while the variable flip angle sequence allows long TE times [33,34]. A multi-spin-echo T2 (MSE-T2), using a 10-echo Carr-Purcell-Meiboom-Gill sequence, was acquired to calculate the T2 value map. Analysis of the images was completed in three modules: muscle segmentation, pre-processing, and biomarker analysis. As a prerequisite, we first segmented the major proximal pelvic limb muscles in the MRI images. All proximal pelvic limb muscles were segmented but only five slices at the midfemur were analyzed and averaged.

For the sake of this study, the biomarker analysis was limited to muscle volumes, T2 mapping values, and several texture analysis features, including a first-order intensity histogram texture feature (entropy) and two high order run length matrix features (short run emphasis (SRE) and run length non-uniformity (RLN)). These texture analysis features were assessed as potential markers of patchy lesions such as necrosis [31,32,35,36]. Based on the mathematical model, we refer to short run emphasis as the Small Lesion Index (SLI) and non-uniformity as the Heterogeneity Index (HI). Both SLI and HI use the run-length matrix method. Compared to histogram-based biomarkers that use intensity data only, the run-length matrix method also takes into account the spatial distribution and intensity of the voxels. A gray-level ‘run’ is defined as a set of consecutive voxels of similar intensity level in a given direction within a predefined similarity range. This is run in a three-dimensional matrix and is intended to detect lumps of hyper-intensity in MRI. To determine overall muscle scores for T2 and the texture features in each group, the proportional muscle volume was considered, so as to calculate a weighted average.

### Cranial sartorius (CS) circumference

We have previously shown that the CS muscle undergoes dramatic hypertrophy in GRMD dogs and that this hypertrophy tracks with postural abnormalities [37]. Accordingly, we use CS circumference measured at surgery during routine biopsy as a surrogate for muscle hypertrophy and associated postural changes in GRMD [24]. Dogs were anesthetized (above), and an incision was made over the cranial aspect of the thigh. In advance of biopsy, the CS muscle was isolated. Nylon suture was placed around the muscle at approximately midsection and tightened so as to snugly encircle the muscle belly. The two ends of the suture were secured with a pair of hemostats and then cut on the muscle side of the hemostat. The length in mm was divided by body mass in kg (mm/kg). An average of two measurements was recorded.

### Histopathologic assessments

For mice muscles, histopathological assessment was performed as earlier described [13]. For canine samples, CS, lateral gastrocnemius, vastus lateralis, and diaphragm muscles were assessed at the end of the 4-month treatment period when dogs were necropsied. Muscle samples were snap frozen in isopentane cooled in liquid nitrogen, and stored at -80°C. A total of 16 dogs (3 wild type, 2 wild type + NBD, 5 untreated GRMD, and 6 GRMD + NBD) were assessed. Serial frozen sections (n = 10 per dog) from each muscle were processed. For each stain, quantitation was performed on three 10 μm sections of each muscle. To determine the degree of inflammation, cells that stained with a canine specific macrophage PM2K antibody were quantitated. Muscle damage was assessed by scoring for IgG-positive myofibers using immunofluorescence, and necrotic foci by hematoxylin and eosin (H&E) staining. Centrally located nuclei (CLN) were quantified on H&E sections to determine the degree of regeneration. Staining was quantitated on an Olympus BX51 microscope with Microsuite Five software (Olympus Soft Imaging Solutions GmbH, Center Valley, PA, USA). A composite score for all four muscles, reflecting muscle injury, inflammation, and regeneration, was determined.

Necropsies were completed on all six of the NBD-treated GRMD dogs and two of the wild type dogs. Sections of kidney, liver, spleen, lung, heart (right and left ventricle), popliteal lymph node, adrenal gland, thyroid, duodenum, large intestine, pancreas, stomach, and cerebrum were collected and fixed in 10% buffered formalin. Tissues were sent to Histo-Scientific Research Laboratories in Frederick, MD, USA, a contract research laboratory where they were processed, embedded in paraffin, sectioned, and stained with H&E. An American College of Veterinary Pathology-certified pathologist evaluated slides.

### Statistics

For the functional and MRI studies, we focused on comparisons between two types of wild type dogs (3 wild type dogs treated with NBD versus 8 wild type natural history dogs) and between two types of GRMD dogs (6 GRMD dogs treated with NBD versus 10 GRMD natural history dogs). For all dogs, MRI scans were obtained at around 6 months of age (171.0 ± 10.9 days). To compare the MRI features between two groups, we carried out a two-sample *t*-test for each muscle. This test is known as Welch’s *t*-test since the two data groups have unequal sample sizes and the group variances are assumed to be unequal [38]. We also compared the functional data on both the natural history (wild type and GRMD) and GRMD dogs treated with NBD. For each dog, we utilized the *t*-test for comparing two groups at each age. For all functional and MRI tests, we applied the FDR method to correct the *P* values [39]. Significance was set at *P* <0.05; trends were reported when *P* <0.2. Histopathologic data were analyzed using an unpaired Student *t*-test. A two-tailed *P* value of <0.05 was considered significant.

## Results

### Establishing a delivery and dosing schedule for NBD in the *mdx* mouse

**Figure 1:**
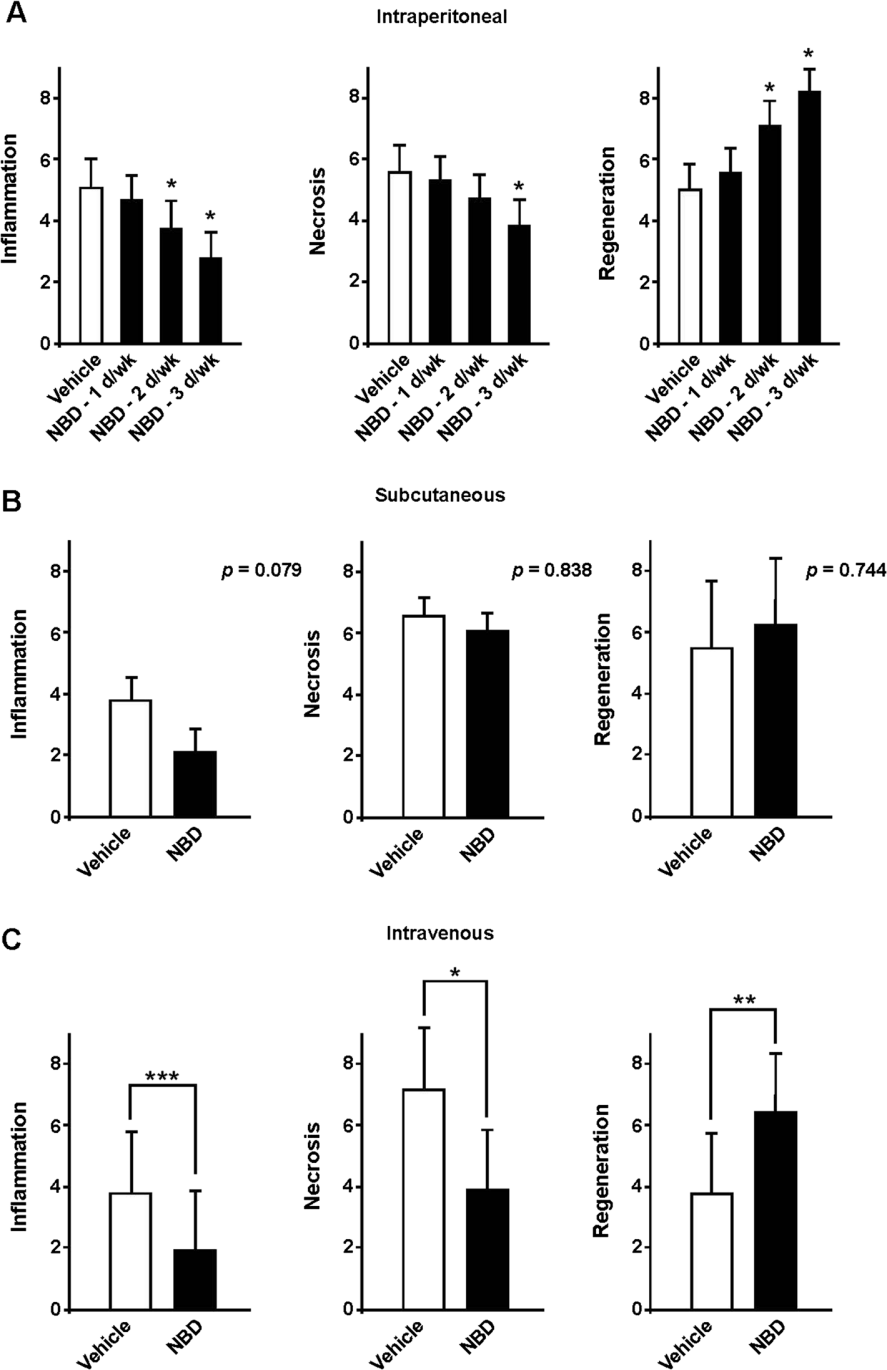
**Establishing a delivery and dosing schedule for NBD in *mdx *mice. (A)***Mdx* mice were treated by IP delivery for a period of 4 weeks with NBD (10 mg/kg) for either 3 days (d), 2 days, or 1 day per week. Quadriceps (Quad) muscles were selected as a representative hind limb muscle from NBD treated *mdx* mice (n = 5 per group). **(B)***Mdx* mice were treated with vehicle or NBD (10 mg/kg), 3× weekly, by SQ delivery, for a period of 4 weeks. Quad muscles were analyzed from NBD and vehicle treated *mdx* mice (n = 10 per group). **(C)***Mdx* mice were treated with vehicle or NBD (10 mg/kg) 3× weekly, by IV delivery for a period of 4 weeks. Quad muscles were analyzed from NBD and vehicle treated mice (n = 5 per group). For all panels, muscle sections (3 per muscle) were either immunostained with the macrophage marker, F4/80, to measure inflammation, or incubated with a fluorescent conjugated immunoglobin (IgG) to assess necrosis, or stained with the embryonic form of myosin heavy chain (eMyHC) to detect regenerative myofibers. Data are expressed as percentage of marker expression per total area. Asterisks indicate *P* = 0.05 (*), *P* <0.01 (**), *P* <0.001 (***).

Previous results in *mdx* and dko mice showed that intraperitoneal (IP) dosing of NBD, 3× per week, was efficacious in improving function in skeletal and/or cardiac muscle and lessening histopathologic lesions of skeletal muscles [13,14]. In addition, we showed that this response was dose dependent, as efficacy was lost when concentrations of NBD were reduced from 10 to 2 mg/kg [13]. To further optimize NBD delivery, we tested whether benefits could be maintained by dosing *mdx* mice at 10 mg/kg at 2× or 1× per week. In comparison to our standard 3× per week schedule, histopathologic improvement was less pronounced with reduced treatment frequencies (Figure 1A). Next, we tested different administration routes since IP delivery is not feasible for DMD patients. While our previous findings showed that IP delivery of NBD was effective in significantly reducing muscle inflammation and necrosis [9,13,40], no such improvements were observed following subcutaneous dosing (Figure 1B). In contrast, delivering NBD by intravenous (IV) dosing for 4 weeks using a VAP, which allowed repeated dosing through a catheter line, resulted in significant histopathologic improvement in *mdx* skeletal muscles (Figure 1C). This suggested that IV delivery might be a suitable route for dosing NBD in larger species. This point was further supported by PK measurements, which showed a dose dependent increase of NBD in the blood of normal mice following single IV injections of the peptide at 2 and 10 mg/kg (Additional file 1: Figure S1A).

### Establishing a treatment paradigm for GRMD dogs

Having established a treatment regimen that provides efficacy of NBD in mice, we next asked whether such therapy could be applied to a larger animal model of DMD. PK studies after IV delivery of NBD to normal beagle dogs showed there was a dose dependent increase of NBD following injections of 2 and 10 mg/kg of peptide (Additional file 1: Figure S1B). This PK profile was comparable to that of mice, suggesting that the murine dosing regimen would extrapolate to dogs. In addition, normal hematology (white blood cell, red blood cell, platelet counts) and serum chemistry (ALT, AST, GGT, bilirubin, creatinine, potassium, creatine kinase) profiles were noted following a single IV injection of NBD in normal dogs (data not shown). Based on our collective data, a preclinical trial in GRMD dogs was initiated with four primary outcomes: skeletal muscle function, MRI of pelvic limb muscles, histopathologic features of skeletal muscles, and safety. Juvenile GRMD (n = 6) and wild type (n = 3) dogs at 2 months of age were treated for 4 months by IV infusions, 3× weekly, at 10 mg/Kg. Results were compared with those collected from untreated GRMD and wild type dogs through a separate, natural history study.

### NBD treatment enhances GRMD muscle function and reduces postural abnormalities

#### ***TTJ force***

We have previously assessed force generated by TTJ flexion and extension in both natural history and preclinical studies [20,23]. Data from a recently completed natural history study were compared with results from NBD-treated GRMD dogs at 6 months of age (Table 1). Force values, corrected for body weight, were used as the primary outcome parameter to be consistent with the prior studies. Presumably owing to changes in instrumentation and methodology [24,25], these natural history data differ somewhat from our previously published results, making direct comparison difficult. Importantly, data from the control natural history and NBD-treated dogs reported here were collected using the same instrumentation and over a similar time frame.

**Table 1:** Phenotypic measures in NBD-treated and untreated GRMD and normal dogs

**Test**	**Normal (Untreated) (n = 8)**		**Normal (NBD-treated) (n = 3)**		**GRMD (Untreated) (n = 10)**		**GRMD (NBD-treated) (n = 6)**	
	**Age (months)**		**Age (months)**		**Age (months)**		**Age (months)**	
	**3**	**6**	**2**	**6**	**3**	**6**	**2**	**6**
Body Mass (kg)	8.46 ± 0.63^2*^	17.80 ± 0.44^1***,2***^	4.99 ± 1.83	21.10 ± 3.15^1*^	7.36 ± 1.29	13.40 ± 2.20^1***^	3.60 ± 0.27	12.70 ± 2.81^1**^
Absolute Tetanic Flexion Force (N)	9.51 ± 1.07^2***^	23.19 ± 2.20^1***,2***^	4.52 ± 1.82	25.75 ± 2.18^1**^	3.85 ± 1.13	7.85 ± 1.98^1***^	2.05 ± 0.58	6.79 ± 1.13^1***^
Body-mass Corrected Tetanic Flexion Force (N/kg)	1.12 ± 0.10^2***^	1.30 ± 0.13^1*,2***^	0.90 ± 0.04^2**^	1.24 ± 0.19	0.53 ± 0.14	0.62 ± 0.18	0.57 ± 0.15	0.55 ± 0.13
Absolute Tetanic Flexion Torque (Nm)	1.08 ± 0.14^2***^	3.22 ± 0.35^1***,2***^	0.42 ± 0.24	3.65 ± 0.43^1**^	0.43 ± 0.14	1.03 ± 0.23^1***^	0.17 ± 0.06	0.89 ± 0.18^1***^
Body-mass Corrected Tetanic Flexion Torque (Nm/kg)	0.13 ± 0.01^2***^	0.18 ± 0.02^1***,2***^	0.08 ± 0.02	0.17 ± 0.02	0.06 ± 0.02	0.08 ± 0.02	0.05 ± 0.01	0.07 ± 0.02^1*^
Absolute Tetanic Extension Force (N)	22.56 ± 2.76^2***^	46.43 ± 8.44^1***,2***^	13.46 ± 3.26	42.17 ± 7.46^1*^	12.73 ± 2.12	18.47 ± 10.11	7.50 ± 2.09	34.30 ± 9.65^1**,2*^
Body-mass Corrected Tetanic Extension Force (N/kg)	2.68 ± 0.38^2**^	2.61 ± 0.49^2**^	2.88 ± 1.10	2.00 ± 0.22	1.80 ± 0.52	1.56 ± 0.92	2.11 ± 0.70	2.70 ± 0.65^2*^
Absolute Tetanic Extension Torque (Nm)	2.55 ± 0.37^2***^	6.44 ± 1.24^1***,2***^	1.20 ± 0.41	6.02 ± 1.38^1*^	1.42 ± 0.23	2.46 ± 1.28	0.62 ± 0.16	4.55 ± 1.49^1**,2*^
Body-mass Corrected Tetanic Extension Torque (Nm/kg)	0.30 ± 0.04^2***^	0.36 ± 0.07^2***^	0.25 ± 0.08	0.28 ± 0.04	0.20 ± 0.05	0.18 ± 0.10	0.21 ± 0.07	0.36 ± 0.08^1*,2**^
ECD (%; 1-10)	13.68 ± 3.97	8.29 ± 2.88^1*,2**^	13.36 ± 2.65	14.47 ± 7.31	13.97 ± 10.52	28.96 ± 18.07	16.12 ± 5.88	32.04 ± 13.46
ECD (%; 1-30)	26.80 ± 5.92^2*^	17.86 ± 5.20^1*,2***^	33.44 ± 10.3	23.25 ± 17.4	36.51 ± 11.49	54.30 ± 14.73^1*^	38.67 ± 10.34	54.48 ± 13.64
TTJ Angle (^o^)	167.9 ± 3.94^2***^	160.9 ± 3.40^1**,2*^	161.7 ± 7.64	158.3 ± 2.08	159.40 ± 3.72	151.30 ± 10.19	157.00 ± 9.80	160.17 ± 8.84
Hip (Resting)	99.50 ± 5.10	104.4 ± 5.76	99.33 ± 1.15	103.0 ± 11.4	103.4 ± 8.96^^^	109.6 ± 8.02	103.7 ± 2.94	100.2 ± 6.01^2*^
Maximum Hip Flexion (^o^)	55.50 ± 8.86	59.00 ± 12.60	48.67 ± 7.77	53.33 ± 10.12	54.40 ± 7.50^^^	68.60 ± 16.15	44.67 ± 5.47	46.33 ± 3.50^2**^
Pelvic Angle (^o^)	43.75 ± 5.12	36.14 ± 5.98^2**^	38.33 ± 1.53	40.33 ± 5.03	40.00^#^	48.30 ± 5.54	41.00 ± 6.81	36.83 ± 5.12^2**^
CS Circumference (mm/kg)	NA	2.09 ± 0.40^2***^	NA	2.30 ± 0.49	NA	4.03 ± 1.04	NA	3.67 ± 0.65

Functional studies, chiefly TTJ tetanic force and pelvic limb joint angles, were assessed to demonstrate therapeutic benefit of NBD in GRMD dogs and potential effects in wild type dogs. N = Newtons; Nm = Newton meters; ECD = Eccentric contraction deficit; CS = Cranial Sartorius; TTJ = tibiotarsal joint.

^^^Data for only five GRMD dogs available at 3 mos.

^#^Data for only one GRMD dog available at 3 mos.

^1^Significantly different from 2/3 to 6 mos, same genotype; ^*^p < 0.05, ^**^p < 0.01; ^***^p < 0.001.

^2^Significantly different from GRMD natural history; ^*^p < 0.05, ^**^p < 0.01; ^***^p < 0.001.

**Figure 2:**
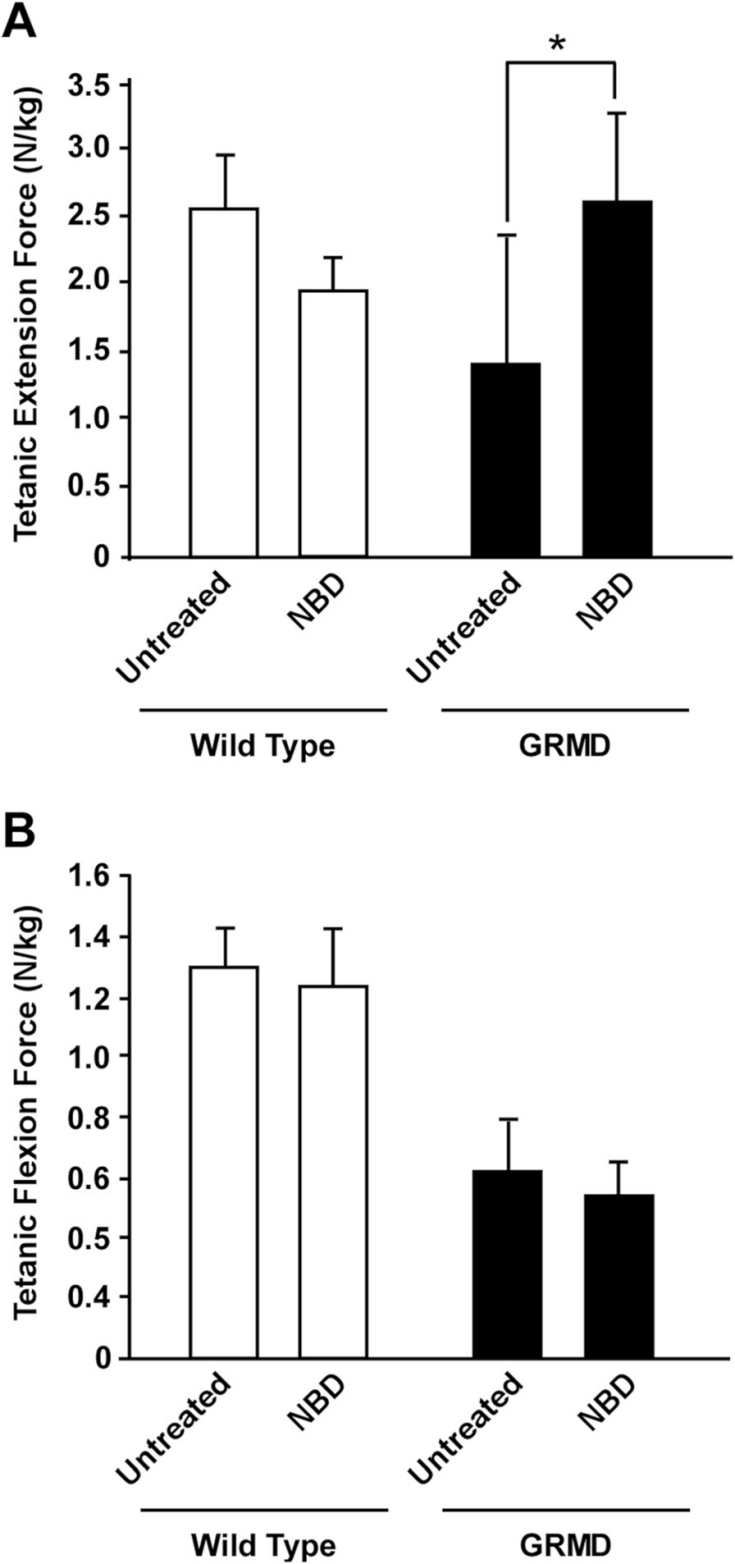
**NBD treatment improves extension force in GRMD dogs.** Wild type and GRMD dogs were administered NBD IV at 10 mg/kg dose, 3× weekly for 4 months. Tibiotarsal joint (TTJ) tetanic extension **(A)** and flexion **(B)** force were measured in NBD-treated wild type and GRMD dogs and compared to untreated wild type GRMD control data from a separate natural history study. Extension force was significantly increased (*P* <0.05 [*]) in NBD-treated GRMD dogs compared to untreated dogs. Flexion force was lower in GRMD dogs treated with NBD but the difference was not significant. White bars represent wild type dogs, while black bars represent GRMD dogs, either untreated or treated with NBD.

GRMD dogs treated with NBD for 4 months exhibited a significant 73% increase in extension force when compared to untreated GRMD dogs (2.70 ± 0.65 vs. 1.56 ± 0.92 N/kg; *P* = 0.038) (Figure 2A, Table 1). In a previous GRMD prednisone trial, treated dogs also had increased extension force [21]. This functional benefit obtained by NBD in GRMD dogs was consistent with that obtained in the *mdx* murine model of DMD [13]. Interestingly, with both prednisone and NBD treatment, body weight corrected flexion force was reduced in treated versus control dogs, although differences did not reach significance. Neither extension nor flexion force differed significantly between wild type control and NBD-treated dogs (Figure 2A and B, Table 1).

#### Eccentric contraction decrement (ECD)

GRMD dogs, like *mdx* mice, exhibit a force decrement with eccentric (lengthening) contractions [26]. We measured the degree of ECD of TTJ flexors while a servomotor simultaneously extended the joint. As with our prior study, ECD in GRMD dogs was higher than that of wild type dogs at 6 months (Table 1). Values in NBD-treated dogs after 10 and 30 contractions (32.0% ±13.5; 54.5% ±13.6) did not differ from those from the natural history GRMD dogs (29.0% ±18.1; 54.3% ±14.7). Thus, our findings indicate that NBD treatment does not stabilize the muscle cell membrane, in contrast to what would be expected with dystrophin transgenes and surrogates [41,42]. These data are consistent with our earlier conclusion in *mdx* mice that protection of dystrophic muscles through NF-κB inhibition does not result from increased membrane stability [9]. The ECD values for control and NBD-treated wild type dogs did not differ (Table 1).

#### ***Joint angles***

Joint angles were measured to determine the severity of contractures and overall postural instability. Proximal pelvic limb joint and postural changes in GRMD dogs appear to contribute to their characteristic plantigrade tarsal stance, just as relative sparing of proximal flexor muscles plays a role in distal limb flexor contractures in DMD [43,44]. Indeed CS muscle circumference corrected for body weight correlates negatively with TTJ angle in GRMD dogs [24]. This suggests that the hypertrophied CS muscle might play a role analogous to iliotibial band tightness in DMD [45].

**Figure 3:**
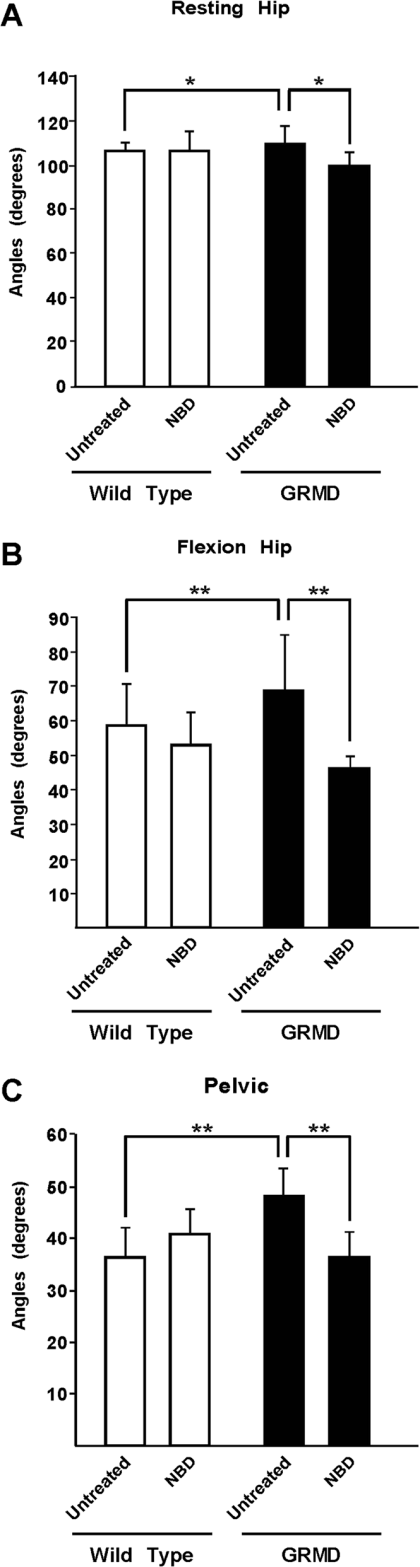
**NBD treatment reduces postural changes in GRMD dogs.** Joint angles were measured to determine if NBD treatment improved postural changes in GRMD dogs. Angles formed by the hip at rest **(A)** and with maximal flexion **(B)** and the line of the pelvis **(C)** were reduced (less restricted) in GRMD dogs treated with NBD compared to untreated controls. *P* <0.05 (*), *P* <0.01 (**). White bars represent wild type dogs, while black bars represent GRMD dogs, either untreated or treated with NBD.

We measured angles at rest and with maximum flexion and extension at the three pelvic limb joints using a standard technique [29]. Resting (Figure 3A, Table 1) and flexion (Figure 3B, Table 1) hip angles were smaller (less restricted) in the NBD treated versus control GRMD dogs. To further characterize postural changes typical of GRMD [20], we measured the pelvic angle formed by the spine and a line drawn between the tuber ischium and the tuber coxae. The pelvic angle values were significantly reduced in NBD treated versus untreated GRMD dogs (Figure 3C, Table 1). Joint angles for control and NBD treated wild type dogs did not differ (Figure 3, Table 1). Taken together, joint angle changes in treated GRMD dogs were consistent with less pronounced postural deformity, which could reflect reduced muscle necrosis/inflammation and an associated reduction in flexor muscle hypertrophy.

### NBD treatment is associated with normalization of MRI features supporting a phenotype of reduced necrosis or inflammation

The potential role of MRI as a biomarker in GRMD has been reported in both natural history [31,32,46-48] and preclinical [49] papers. Various parameters have been assessed, with T2 signal intensity used most commonly as a feature of either increased fluid or fat. T2 signal was decreased in GRMD dogs treated systemically with morpholinos to restore dystrophin expression compared to age-matched untreated dogs in one study [49], supporting a role for MRI as a biomarker in preclinical studies. We recently completed an MRI natural history study of proximal pelvic limb muscles of GRMD and wild type dogs over the first 9 to 12 months of age [32]. The most striking differences were seen between 3 and 6 months, indicating that biomarkers assessed over this period would most likely reflect therapeutic benefit. Muscle volume and the texture analysis biomarker, run length non-uniformity (termed Heterogeneity Index [HI] here), differed most markedly. The biceps femoris, semitendinosus, and CS muscles demonstrated the greatest differential progression in GRMD versus wild type dogs [32].

Based on these natural history data, we assessed MRI at 6 months as an additional outcome measure for NBD treatment of GRMD dogs, focusing on muscle volume, T2 mapping values, and two texture analysis features, HI and short run emphasis (termed small lesion index (SLI) here). Texture analysis features were included because they take into account the spatial distribution of lesions, potentially highlighting the patchy nature of necrosis.

**Figure 4:**
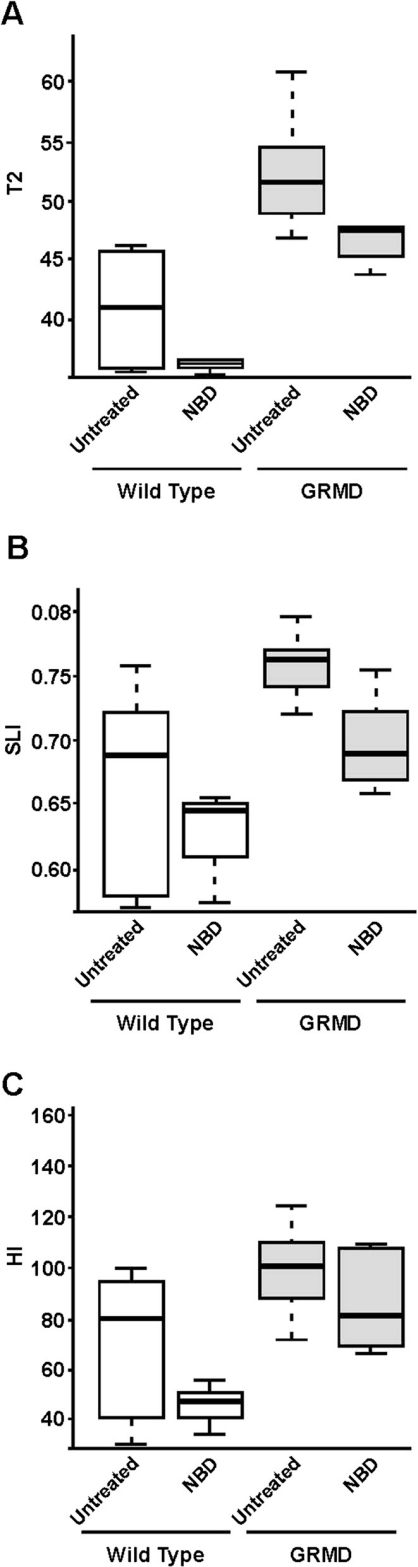
**MRI values differed between NBD-treated and untreated GRMD and wild type dogs.** Overall weighted average muscle scores for T2 mapping values **(A)** and the two texture analysis features, short run emphasis (Small Lesion Index; SLI) **(B)** and run length non-uniformity (Heterogeneity Index; HI) **(C)** were lower in wild type versus GRMD dogs. Each trended even lower with NBD treatment. The median and upper and lower quartiles for each value are identified. White bars represent wild type dogs, while gray bars represent GRMD dogs, either untreated or treated with NBD.

For T2 mapping, there was a strong trend for lower values in all muscles considered as a whole in NBD-treated GRMD dogs (47.3 ± 3.10) when compared to untreated GRMD controls (52.3 ± 4.52) (*P* = 0.092) (Figure 4A; Table 2). Interestingly, NBD treatment also lowered T2 values in wild type dogs (35.9 ± 0.47) compared to untreated wild type (40.8 ± 5.21) controls (*P* = 0.076). When T2 mapping values for individual muscles were assessed, the greatest effect was in the semitendinosus, biceps femoris, and the vastus lateralis and intermedius heads of the quadriceps femoris, all having *P* values <0.1.

**Table 2:** MRI T2 and texture analysis features in NBD-treated and untreated GRMD and wild type dogs

**Muscle**	**Normal (Untreated) (n = 8)**			**Normal (NBD-treated) (n = 3)**			**GRMD (Untreated) (n = 10)**			**GRMD (NBD-treated) (n = 6)**		
	**T2**	**SLI**	**HI**	**T2**	**SLI**	**HI**	**T2**	**SLI**	**HI**	**T2**	**SLI**	**HI**
Cranial Sartorius	49.40 ± 9.01	0.79 ± 0.09	66.80 ± 18.58	47.72 ± 6.86	0.74 ± 0.04	59.82 ± 29.72	49.87 ± 6.44	0.79 ± 0.05	74.01 ± 27.15	49.40 ± 9.01	0.79 ± 0.05	59.82 ± 14.91
Caudal Sartorius	53.87 ± 9.88	0.72 ± 0.12	27.96 ± 15.47	54.02 ± 4.79	0.81 ± 0.04	35.21 ± 9.28	59.71 ± 7.02	0.83 ± 0.07	56.90 ± 30.08	53.87 ± 9.88	0.83 ± 0.05	35.21 ± 14.92
Vastus Lateralis	40.67 ± 5.84	0.62 ± 0.07	66.18 ± 28.71	36.60 ± 1.37	0.63 ± 0.12	52.92 ± 23.51	49.84 ± 5.35	0.75 ± 0.07	69.42 ± 18.85	40.67 ± 5.84^^^	0.68 ± 0.04^^^	52.92 ± 27.20
Vastus Intermedius	41.91 ± 5.91	0.66 ± 0.19	42.44 ± 21.75	36.03 ± 0.73^^^	0.58 ± 0.06	59.10 ± 9.05	52.62 ± 6.33	0.75 ± 0.06	69.83 ± 39.19	41.91 ± 5.91^^^	0.68 ± 0.09	59.10 ± 27.19
Vastus Medialis	42.74 ± 5.65	0.72 ± 0.11	63.20 ± 23.57	35.80 ± 0.94^^^	0.64 ± 0.06	52.55 ± 9.41	53.60 ± 7.16	0.82 ± 0.06	41.13 ± 14.73	42.74 ± 5.65	0.77 ± 0.09	52.55 ± 22.29
Rectus Femoris	37.23 ± 4.72	0.70 ± 0.06	55.30 ± 29.46	33.30 ± 2.41	0.64 ± 0.03	55.42 ± 3.46	49.44 ± 5.71	0.78 ± 0.06	57.02 ± 23.66	37.23 ± 4.72	0.68 ± 0.07^^^	55.42 ± 21.95
Biceps Femoris	40.20 ± 4.91	0.67 ± 0.06	91.36 ± 35.35^^^	34.94 ± 0.40	0.62 ± 0.07	87.50 ± 11.65	50.13 ± 4.78	0.76 ± 0.06	137.3 ± 40.91	40.20 ± 4.91^^^	0.68 ± 0.06^^^	87.50 ± 48.42
Semimembranosus	39.42 ± 4.86	0.63 ± 0.08	49.75 ± 20.32	35.24 ± 1.17	0.63 ± 0.10	78.15 ± 20.38	52.28 ± 4.55	0.71 ± 0.07	121.4 ± 59.04	39.42 ± 4.86	0.68 ± 0.06	78.15 ± 33.31
Semitendinosus	40.41 ± 4.97	0.61 ± 0.19	44.65 ± 21.35	36.59 ± 2.18	0.61 ± 0.13	81.43 ± 38.69	56.09 ± 6.02	0.77 ± 0.07	128.1 ± 42.03	40.41 ± 4.97^^^	0.67 ± 0.08^^^	81.43 ± 35.00
Adductor	39.52 ± 4.76	0.67 ± 0.11	87.90 ± 42.89	34.69 ± 0.91^^^	0.64 ± 0.00	116.18 ± 7.79	50.49 ± 5.19	0.72 ± 0.05	84.12 ± 33.68	39.52 ± 4.76	0.72 ± 0.02	116.2 ± 22.37
Gracilis	45.91 ± 3.50	0.63 ± 0.13	59.83 ± 38.30	38.27 ± 0.93^**^	0.62 ± 0.06	58.90 ± 24.51	58.56 ± 5.19	0.78 ± 0.05	104.2 ± 34.65	45.91 ± 3.50	0.70 ± 0.06^^^	58.90 ± 30.66
All	40.81 ± 0.47	0.66 ± 0.08	71.06 ± 27.89	35.90 ± 0.47^^^	0.63 ± 0.04	47.12 ± 10.24	52.28 ± 4.52	0.75 ± 0.04	103.47 ± 22.40	47.34 ± 3.10^^^	0.70 ± 0.04^^^	86.01 ± 18.28

MRI values are shown for individual thigh muscles. A composite value for all muscles combined is also shown. Significantly different or trending towards different from untreated control, same genotype; ^**^indicates statistical difference from untreated control, same genotype p < 0.01; ^^^indicates trending towards different from untreated control, same genotype p < 0.10.

Of the texture analysis features, SLI for all muscles considered together trended towards being higher in the untreated (0.75 ± 0.04) versus NBD-treated (0.70 ± 0.04) GRMD dogs (*P* = 0.0572) (Figure 4B; Table 2), in keeping with more pronounced patchy lesions, such as necrosis, that would disrupt the pixel run lengths of homogeneous normal muscle. The pattern of individual muscle involvement largely paralleled that of the T2 map, with the semitendinosus, biceps femoris, gracilis, rectus femoris, and vastus lateralis all having *P* values <0.1. The HI feature of all muscles taken together was also higher in untreated GRMD dogs (103.5 ± 22.4) compared to those treated with NBD (86.0 ± 18.3) (*P* = 0.229) (Figure 4C, Table 2). On the other hand, CS texture features did not distinguish treated and control GRMD dogs, with T2 and SLI values being essentially identical and those for HI showing only a modest insignificant lowering in treated dogs. This reflects the differential disease effect evident in the CS [32,37]. Values for untreated wild type dogs (SLI, 0.66 ± 0.08; HI, 71.1 ± 27.9) were lower than those with GRMD and even lower in the NBD-treated group (SLI, 0.63 ± 0.04; HI, 47.1 ± 10.2) (SLI, *P* = 0.726; HI, *P* = 0.205). Values for T2, SLI, and HI tracked with one another, pointing towards shared underlying lesions (Table 2). Taken together, the T2, SLI, and HI results are compatible with an anti-inflammatory effect of NBD and an associated reduction in fluid accumulation and necrosis.

### NBD treatment improves histopathological lesions of GRMD muscles

**Figure 5:**
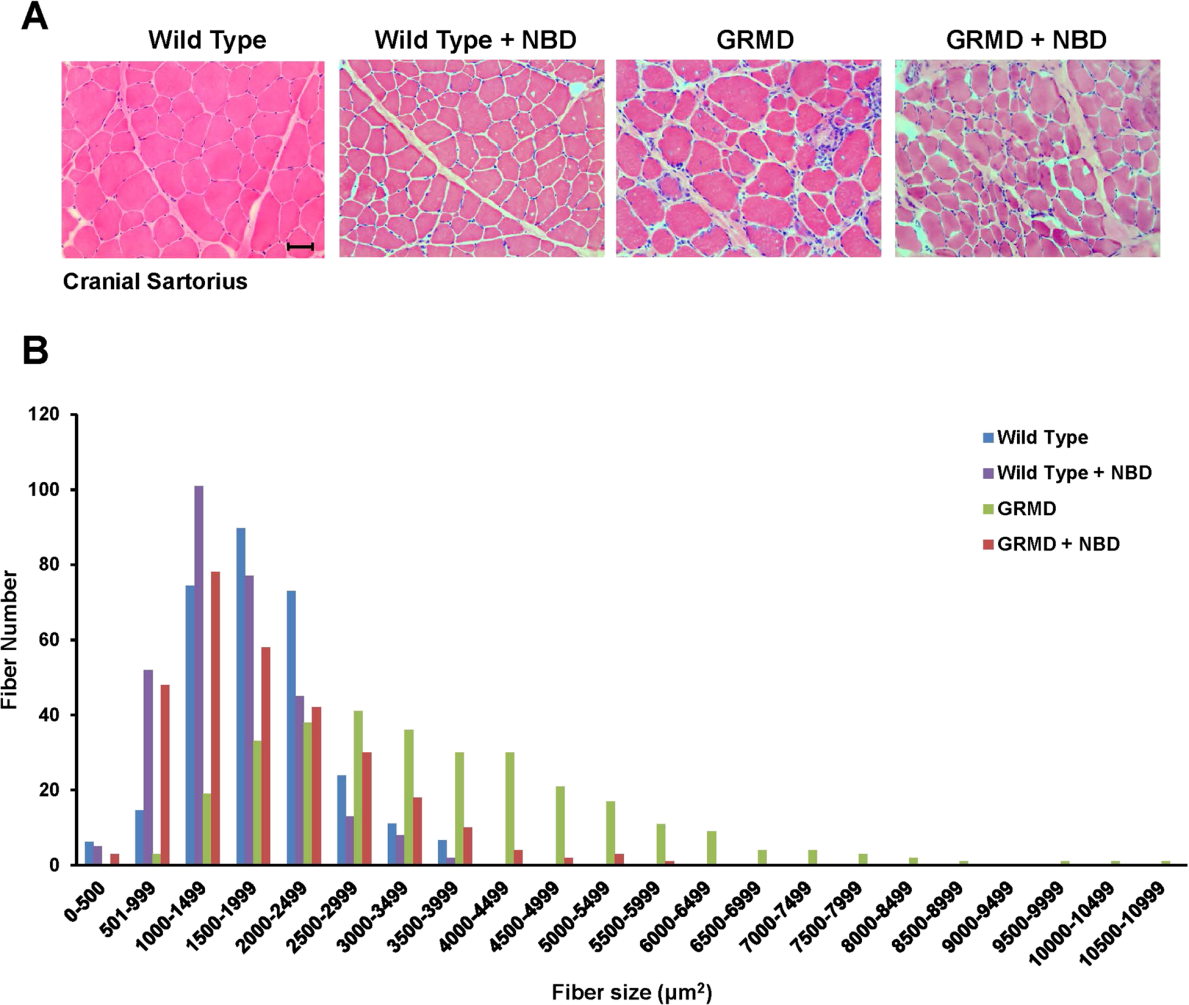
**NBD treatment reduces damage-associated hypertrophy of CS muscles.** Sections from the CS muscle were prepared and stained by H&E. **(A)** Representative sections at 20× magnification are shown from wild type dogs, wild type dogs treated with NBD, GRMD dogs, and GRMD dogs treated with NBD. Scale bar = 50 μM. **(B)** Fiber diameter measurements were made from wild type (n = 2; 600 fibers), wild type treated with NBD (n = 2; 600 fibers), GRMD (n = 5; 1,500 fibers), and GRMD dogs treated with NBD (n = 6; 1,800 fibers).

For pathologic studies, we were particularly interested in the CS muscle, as it undergoes early necrosis followed by true hypertrophy and subsequent fibrosis in GRMD [37]. The degree of hypertrophy correlates with TTJ force measurements and joint angles, and generally tracks with a more severe phenotype [24]. Accordingly, we included CS circumference at 6 months of age as an endpoint measure to determine the efficacy of NBD. Consistent with MRI findings, untreated GRMD dogs had a larger CS circumference (4.03 ± 1.04) compared to wild type controls (2.09 ± 0.40) (*P* <0.001) (Table 1). CS size was reduced in NBD-treated (3.67 ± 0.65) vs. untreated GRMD dogs, but this difference was not significant (*P* = 0.408). The hypertrophic response in CS muscles was clearly observable on measurements of myofiber size in a subset of untreated GRMD (n = 5, 1,500 fibers) versus wild type (n = 2, 600 fibers) dogs (Figure 5A and 5B). Treatment with NBD profoundly reduced myofiber size by 46% (GRMD + NBD, n = 6, 1800 fibers) (*P* <0.05) compared to untreated GRMD muscles.

**Figure 6:**
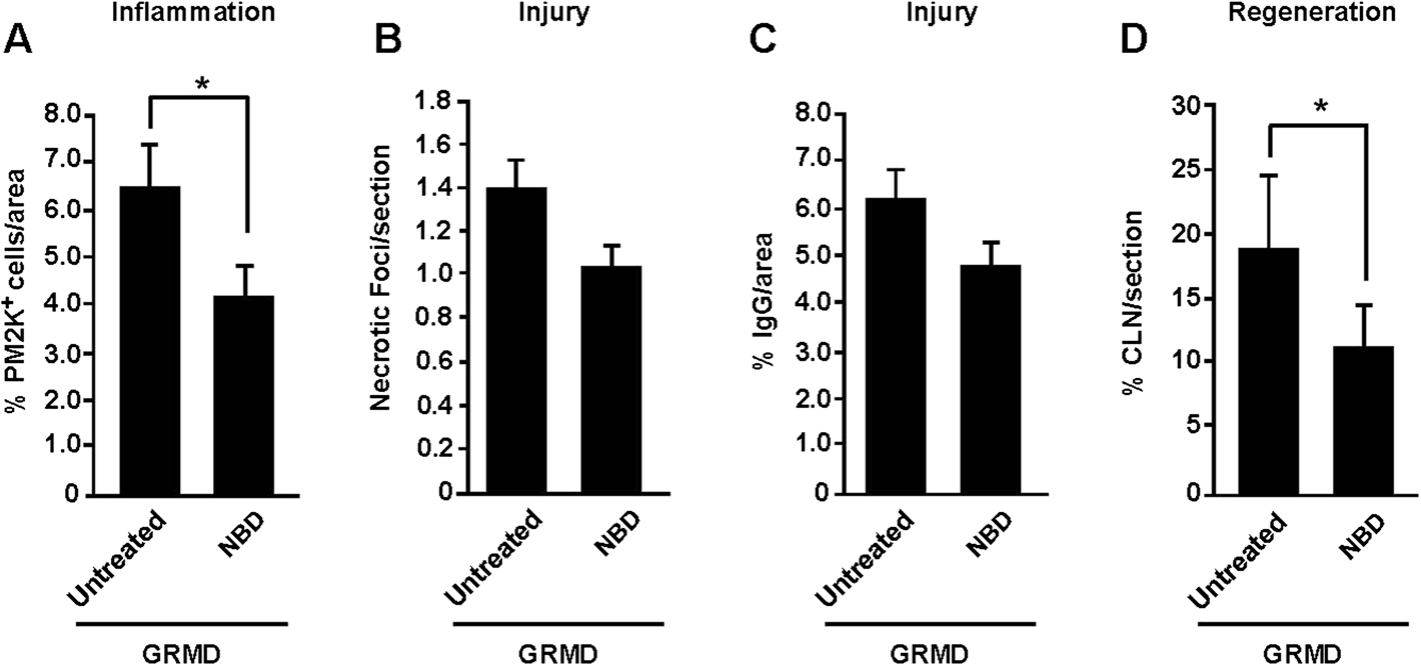
**NBD treatment improves the histopathological changes of GRMD cranial sartorius muscles.** Muscle sections from the cranial sartorius in GRMD and GRMD dogs treated with NBD were analyzed for different histopathology markers including **(A)** PM2K^+^ macrophages, as an index of inflammation, **(B, C)** necrotic foci and IgG cytoplasmic localization, as markers of muscle injury, respectively, and **(D)** central nucleation, as a marker of muscle regeneration. Asterisks indicate *P* < 0.05 (*).

We next determined whether NBD mitigated inflammation. NBD treatment significantly reduced the number of PM2K-positive macrophages by 34% (*P* <0.05) in the CS muscle compared to untreated GRMD dogs (Figure 6A, Table 3). Necrosis and IgG-positive myofibers were also reduced by 25% and 22%, respectively, in NBD-treated dogs, but both of these indices only trended toward significance (*P* = 0.14) (Figure 6B, 6C, Table 3). Centrally located nuclei (CLN) were assessed to gauge regeneration and were reduced by 43% (*P* <0.05) in NBD treated versus untreated GRMD dogs (n = 5) (Figure 6D, Table 3). This reduced regenerative response is consistent with less pronounced necrosis and inflammation with NBD treatment, but also implies that NF-κB inhibition does not effectively enhance satellite cells to potently promote regeneration in dogs as previously observed in mice [9,13,15,16]. Histopathological lesions were negligible in NBD treated versus untreated wild type dogs (data not shown). Analogous results with GRMD dogs were seen in three other muscles (lateral head of the gastrocnemius, vastus lateralis, and diaphragm). Composite scores for all four muscles showed that NBD treatment reduced the histopathological lesions of GRMD muscles by 35.5% (Table 3; *P* <0.05, unless indicated). This protective histopathological effect of NBD is in line with functional responses and MRI analysis discussed above. We have reported similar favorable histopathological protection against injury in the *mdx* model of DMD [9,13].

**Table 3:** Composite analysis of histopathology markers in NBD-treated and untreated GRMD dogs

	**Cranial sartorius**	**Lateral gastroc**	**Vastus lateralis**	**Diaphram**	**Average rescue**
Inflammation	34	40	31	37	35.5
Necrosis (Foci,IgG)	25, 22 (p = 0.14)	34, 35	35, 32	37, 32	35.3, 30.3
Regeneration	43	35	44	41	40.8
			Final Histological Score of Improvement		35.5

Histological markers of inflammation, necrosis, and regeneration were quantitatively assessed from sections prepared from 4 muscle groups (cranial sartorius, lateral gastrocnemius, vastus lateralis, and diaphragm). Percent differences between GRMD and GRMD + NBD treated dogs were calculated and an average value for each histological marker was determined for each muscle group. A final composite average from each of these values were also calculated to represent the overall percent of improvement from NBD treatment with regards to reducing inflammation, necrosis, and regeneration. For all values except where indicated, *p* < 0.05.

### NBD treatment was not associated with biochemical or hematologic changes

In addition to efficacy, we were interested in examining the safety profile of NBD, since long-term dosing in any animal model had yet to be performed. Blood samples at pre-dose, mid dose, and terminal dose were obtained for hematology and serum chemistry analysis. Results from these evaluations showed no adverse effects in NBD treated wild type or GRMD dogs (Additional file 2: Figure S2 and Additional file 3: Figure S3).

### Infusion reactions were seen in NBD-treated wild type and GRMD dogs

After approximately 1 month of treatment, both wild type and GRMD dogs developed infusion reactions of variable severity in their response and duration. Many of these signs were consistent with vasodilation/hypotension associated with IgE-induced type 1 hypersensitivity reactions in dogs, as seen with reactions to proteins in certain vaccines [50,51]. Blood pressure measured during administration of the compound was decreased in association with some infusion reactions. NBD was initially dosed over a period of 10 min. With the onset of reactions, the perfusion time was extended to 30 min and dogs were pretreated with diphenhydramine (Benadryl; 2 mg/kg SQ). This seemed to partially reverse, but did not completely eliminate the reactions. More severely affected dogs were treated with intravenous fluids and diphenhydramine at the onset of the reactions with some improvement.

**Figure 7:**
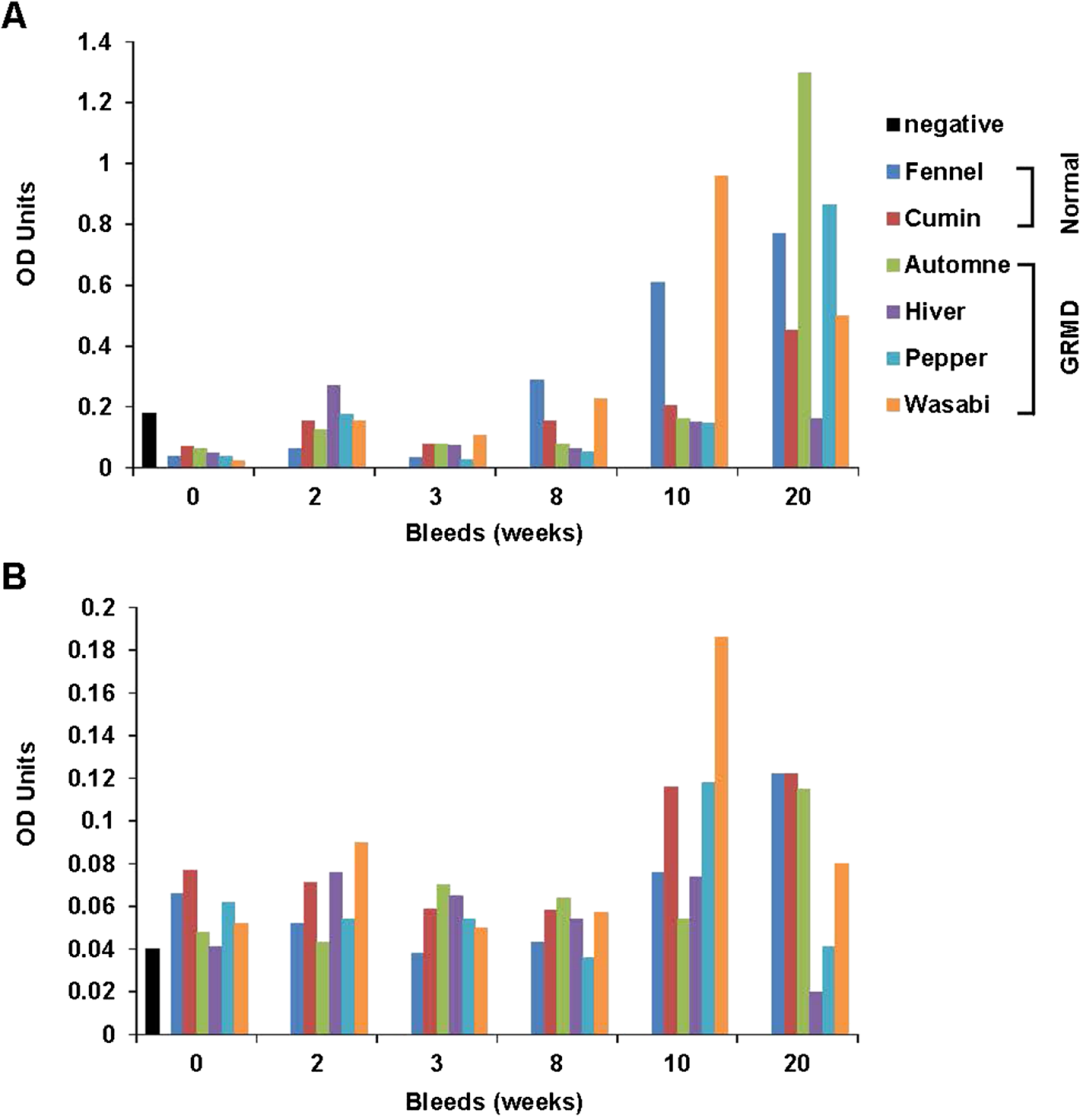
**Levels of immunoglobulins are increased in NBD-treated dogs.** Serum was collected as indicated in weeks during the 4 month trial of NBD treatment on wild type (n = 2) and GRMD (n = 4) dogs. ELISAs were then performed to measure levels of **(A)** IgG and **(B)** IgE compared to naïve canine serum used as a negative control. Values are shown for individual NBD-treated wild type and GRMD dogs. The black bar serves as a negative control.

Although the mechanism of these infusion reactions is not clear, we felt they might be related to an immune response. Therefore, serum from NBD treated dogs was assayed by ELISA for anti-NBD IgG and IgE antibodies. Levels of IgG, and to a lesser extent IgE, increased over time with repeated IV administration of NBD during the 4-month treatment period (Figure 7A and 7B) in some of the GRMD and wild type dogs. Necropsy analysis demonstrated histopathological changes related to NBD, seen principally in the spleen and lungs, and consistent with antigenic stimulation and hypersensitivity [52,53].

In summary, our data support that NBD can be delivered to GRMD dogs over a 4-month period with improved phenotypic outcome and no hematologic or blood chemical abnormalities. However, infusion reactions signify a potential immune response to the peptide.

## Discussion

As with other genetic diseases, treatment strategies for DMD are proceeding on two tracks, one directed at achieving a cure through genetic or cellular approaches, and the other at reducing secondary effects of dystrophin deficiency such as inflammation and fibrosis [54,55]. The use of glucocorticoids, which represents the current standard of care for DMD, is an example of the second treatment strategy. While prednisone and deflazacort delay the clinical progression of DMD, as documented by various outcome parameters [4,5], there are numerous side effects [6]. Accordingly, complementary and alternative forms of therapy, including compounds that inhibit the classical NF-κB signaling pathway, are being sought [16,56-60]. To extend our prior work showing benefit of NBD in the *mdx* and dko mouse models of DMD [13,14], we were motivated to determine whether NBD would have analogous benefits in GRMD dogs.

Results of our functional testing in NBD-treated GRMD dogs showed a substantial increase in extension force and a statistically insignificant paradoxical decrease in flexion force. The increase in extension force is particularly meaningful, since the current gold standard measure for DMD clinical trials is to demonstrate functional benefit. Our ability to collectively achieve such a benefit in GRMD dogs, as well as in *mdx* diaphragm, [9,13] and dko hearts [14], supports the pre-clinical efficacy of NBD. Due to interspecies differences among mice, dogs, and humans, it is difficult to say exactly how much functional improvement in animal models would be needed to increase muscle strength or quality of life in a DMD patient. However, the significant functional responses previously seen in NBD-treated rodent models (*mdx* diaphragm, 43% improvement [13]; dko heart, improved back to wild type [14]), and now in a canine model (GRMD, pelvic limb, 73%), suggest that similar benefits might translate to DMD patients. It is noteworthy that to date, no pharmacologic agent has demonstrated the level of functional efficacy in skeletal and cardiac muscles of mouse and dog DMD models that we achieved with NBD.

Treated dogs also had improved histopathological indices for inflammation and necrosis. We have previously speculated that a trend towards reduced flexion force in GRMD dogs treated with prednisone may reflect a reduction in necrosis that would otherwise lead to functional flexor muscle hypertrophy [21]. A similar trend towards reduced flexion force, as well as normalization of other features of muscle hypertrophy such as CS myofiber size and postural changes, were seen in NBD treated GRMD dogs. Finally, beneficial functional and histopathological features were reinforced by findings on MRI. As expected, the level of eccentric contraction decrement did not differ between NBD treated and control GRMD dogs, reinforcing the fact that NBD’s therapeutic benefit lies in its ability to reduce inflammation rather than restore muscle membrane stability.

One histopathological feature that was not consistent between NBD treated *mdx* mice versus GRMD dogs was the regenerative response to muscle injury. In *mdx* mice, NBD treatment caused a significant increase in muscle regeneration (Figure 1 and [9,13]), in line with earlier findings that NF-κB functions as a negative regulator of skeletal myogenesis [61]. Selective genetic ablation of the NF-κB signaling pathway in *mdx* muscles led to increases in satellite cells, suggesting that constitutive activation of NF-κB in dystrophic muscles functions to repress the regenerative capacity of muscle stem cells [9]. Similar conclusions were reached in studies where NF-κB signaling was ablated by stem cell replacement or gene therapy [15,16]. It is unclear why NBD did not provide this same benefit to GRMD dogs. One possibility, given the development of IgG antibodies, is that NBD could have been neutralized in the later portion of the study, masking a potential enhanced regenerative response. Alternatively, species differences could play a role. *Mdx* mice undergo continual muscle regeneration throughout their lifespan. In contrast, muscle regeneration in dystrophic dogs and DMD patients diminishes over the progression of the disease, potentially due to their shorter telomere length [62] and lower telomerase activity [63]. Thus, it is likely that *mdx* satellite cells are more easily stimulated to expand than canine or human cells. Perhaps, as discussed above in the context of the reduced level of hypertrophy, the relative reduction of necrosis in NBD treated dogs also may have led to a less pronounced regenerative response.

As with any pre-clinical treatment trial in an animal model, we were particularly interested in the safety profile of our product. Notably, NBD treatment in the murine models of DMD [9,13,14] and in numerous other mouse and rat models of disease associated with NF-κB signaling had not reported any side effects. In our current 4-month GRMD NBD trial, several dogs demonstrated infusion reactions characterized by features of vasodilation and hypotension that appeared after about a month of treatment. Although the mechanisms of action for these responses are not clear, signs were indicative of hypersensitivity reactions [50,51]. Some treated dogs also had IgE and IgG antibodies reactive to NBD, lending further credence to a hypersensitivity reaction. Dogs have been used to model hypersensitivity reactions in humans [64] and there is considerable overlap in mast cell function between the two species [65]. It is also possible that immunoglobulins could have resulted from foreign sequences in NBD, as the human version used for these studies differs from that of dogs by two amino acids at the amino terminal end [66]. Safety studies testing a dog version of NBD are currently underway. It is noteworthy that a distinct infusion reaction was recently described in dogs with lymphoma that received a single injection of NBD at 0.5 and 1 mg/Kg [67]. A selective number of dogs developed moderate hypertension soon after the administration of NBD that resolved without treatment. Curiously, this reaction is opposite of the hypotension we observed in this study.

Because NBD-mediated responses seen in normal and diseased dogs may be linked to an immune reaction, efforts are underway to initiate formal pre-clinical toxicology studies in non-human primates, whose immune system more closely resembles man compared to dogs. In a 28-day repeat dosing non-GLP study in non-human primates, systemic infusion responses reported here in dogs were not observed (unpublished observations). However, longer treatment trials with escalating drug doses will be required to draw definitive conclusions on interspecies reactions to NBD treatment. Collectively, the phenotypic benefits seen with NBD in our study are encouraging given the small list of pharmacologic agents that have been tested to date in larger DMD animal models.

## Conclusions

In this study we show that administration of the small peptide inhibitor NBD improves pelvic limb function and ameliorates skeletal muscle histopathological lesions in GRMD dogs. These findings are consistent with earlier findings reported in *mdx* mice, and together suggest that NBD peptide therapy may be a realistic treatment option for DMD.

## Competing interests

The authors declare that they have no competing interests.

## Authors’ contributions

JMP assisted in designing the experiments; JMP, JNK, DJB, WK, JRB, and JLD shared in performing the experiments; ZF, JW, MA, HZ, and MS shared in the statistical analysis; and JNK and DCG shared in designing and directing the overall study. All authors read and approved the final manuscript.

## Supplementary Material

Additional file 1: Figure S1 IV dosing of NBD exhibits a comparable pharmacokinetic profile in mice and dogs. Plasma was prepared from each blood sample collected at 0, 0.08, 0.25, 0.5, 1, 2, 4, 8, and 24 h following IV dosing with NBD at 2 and 10 mg/Kg in normal mice (**A**, n = 3/time point) and beagle dogs (**B**, n = 3/group). Click here for file

Additional file 2: Figure S2 Hematologic changes in NBD-treated dogs. Serum was prepared from blood samples obtained at indicated time points prior to and following dosing with NBD in wild type and GRMD dogs. Serum samples were analyzed for complete blood counts (CBC). The graphs show results for white blood cells, red blood cells, and platelet counts. Click here for file

Additional file 3: Figure S3 Clinicopathologic changes in NBD-treated dogs. Serum was prepared from blood samples obtained at indicated time points prior to and following dosing with NBD in wild type and GRMD dogs. Serum samples were analyzed for ALT, AST, GGT, bilirubin, creatinine, potassium, and creatine kinase. Click here for file
