# Supplementary figures and images for: NBD delivery improves the disease phenotype of the golden retriever model of Duchenne muscular dystrophy
Source: Skelet Muscle. 2014 Oct 23;4:18. doi: 10.1186/2044-5040-4-18 (PMC4364341; doi:10.1186/2044-5040-4-18)

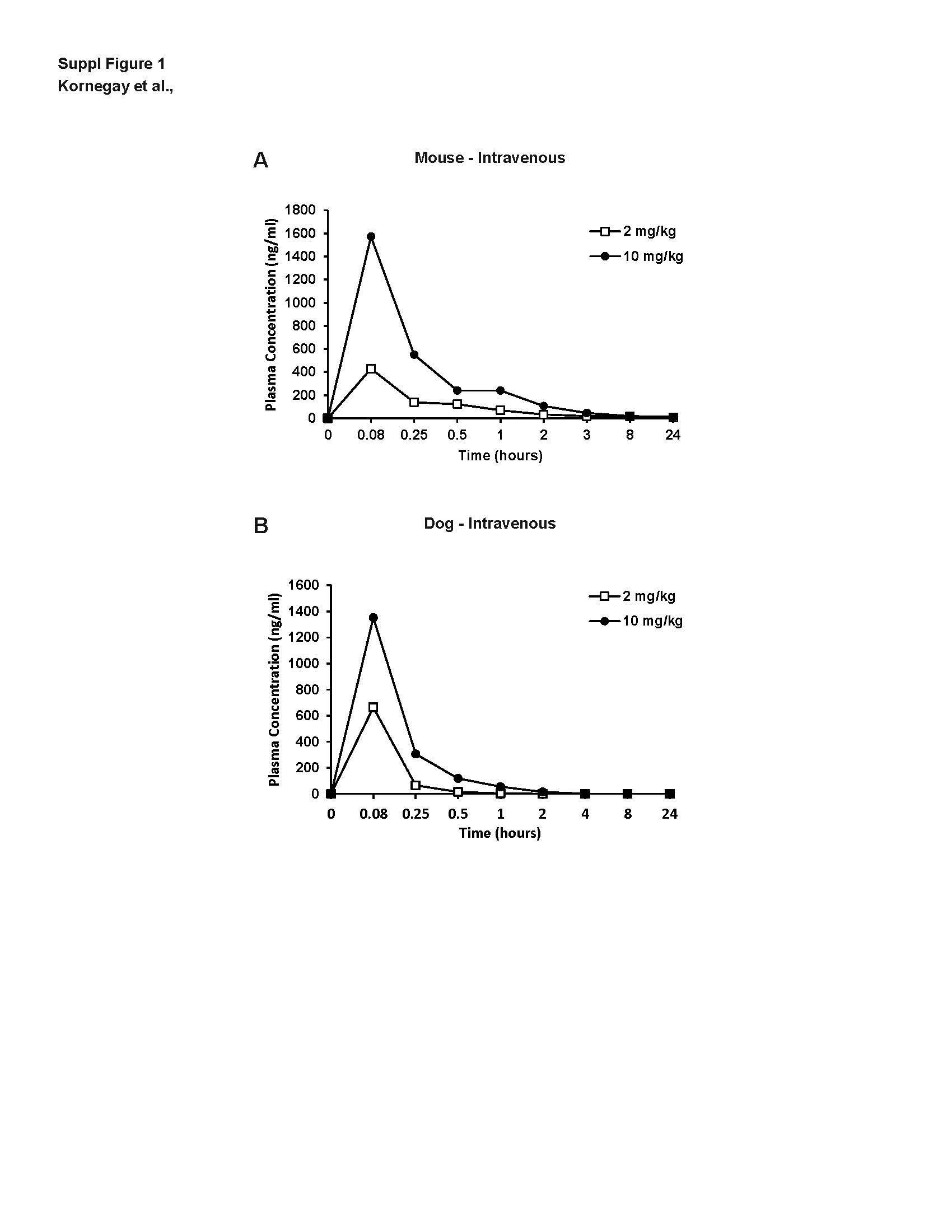

Supplement: Additional file 1: Figure S1 — IV dosing of NBD exhibits a comparable pharmacokinetic profile in mice and dogs. Plasma was prepared from each blood sample collected at 0, 0.08, 0.25, 0.5, 1, 2, 4, 8, and 24 h following IV dosing with NBD at 2 and 10 mg/Kg in normal mice (A, n = 3/time point) and beagle dogs (B, n = 3/group). [file 2044-5040-4-18-S1.jpeg]

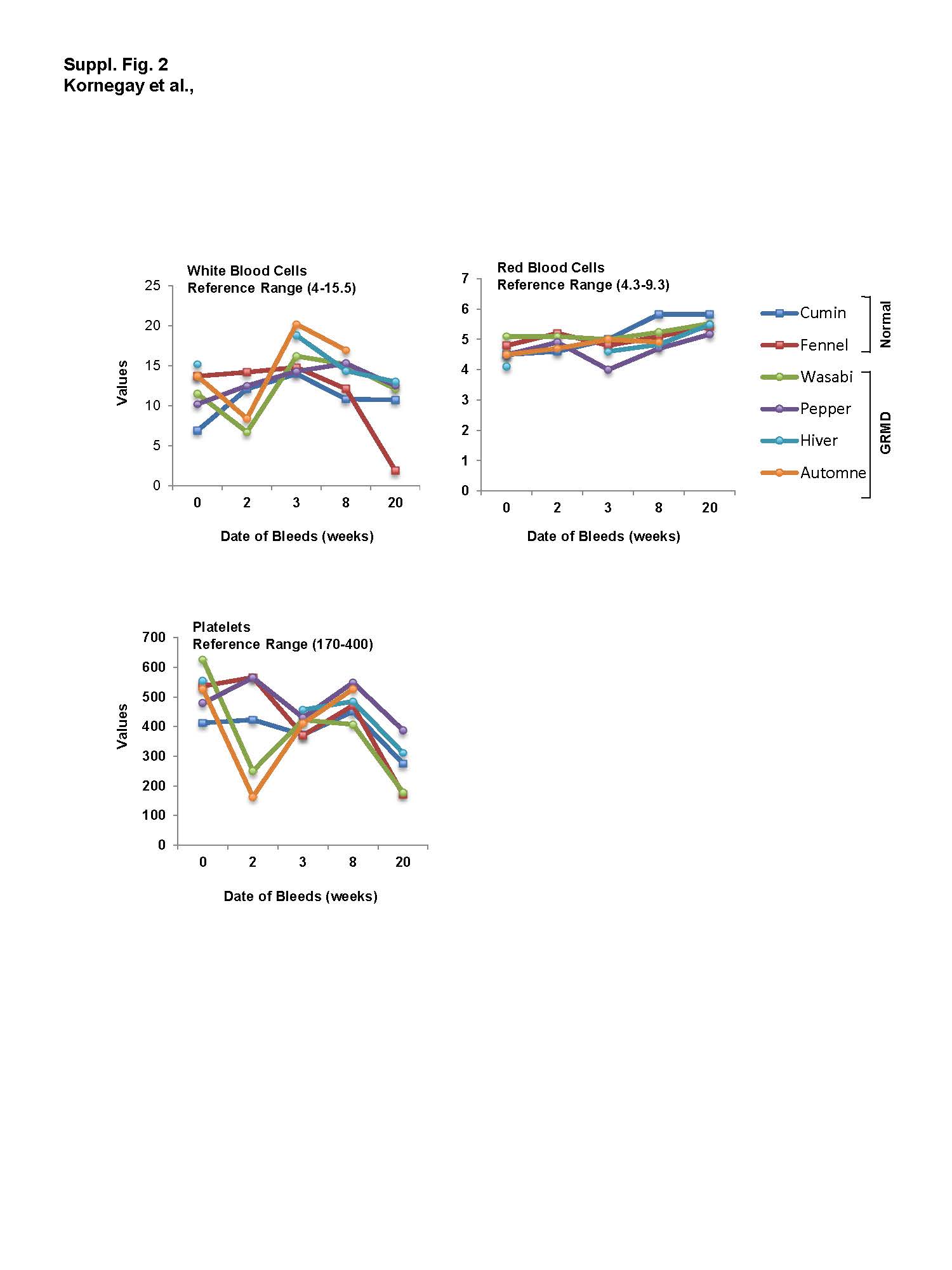

Supplement: Additional file 2: Figure S2 — Hematologic changes in NBD-treated dogs. Serum was prepared from blood samples obtained at indicated time points prior to and following dosing with NBD in wild type and GRMD dogs. Serum samples were analyzed for complete blood counts (CBC). The graphs show results for white blood cells, red blood cells, and platelet counts. [file 2044-5040-4-18-S2.jpeg]

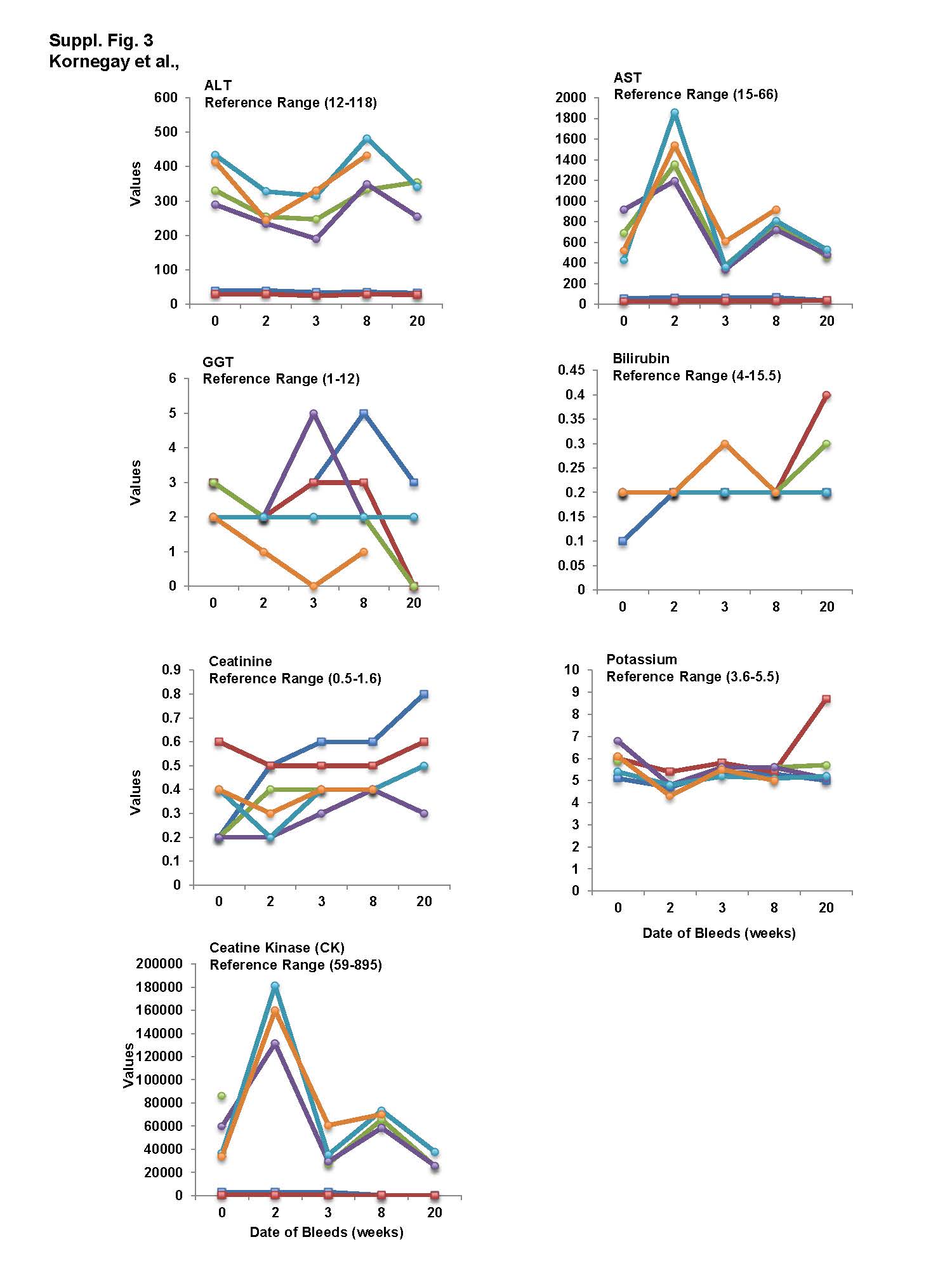

Supplement: Additional file 3: Figure S3 — Clinicopathologic changes in NBD-treated dogs. Serum was prepared from blood samples obtained at indicated time points prior to and following dosing with NBD in wild type and GRMD dogs. Serum samples were analyzed for ALT, AST, GGT, bilirubin, creatinine, potassium, and creatine kinase. [file 2044-5040-4-18-S3.jpeg]
